# Assessing health disparities in breast cancer incidence burden in Tennessee: geospatial analysis

**DOI:** 10.1186/s12905-021-01274-9

**Published:** 2021-05-03

**Authors:** Bonita Salmeron, Lohuwa Mamudu, Xiaohui Liu, Martin Whiteside, Faustine Williams

**Affiliations:** 1 Division of Intramural Research, National Institute on Minority Health and Health Disparities, Bethesda, MD USA; 2 Department of Mathematics and Statistics, University of South Florida, Tampa, FL USA; 3 Tennessee Department of Health, Office of Cancer Surveillance, Nashville, TN USA

**Keywords:** Breast cancer, Health disparities, Geographic information system, Appalachian Tennessee, Non-appalachian Tennessee

## Abstract

**Background:**

Tennessee women experience the 12th highest breast cancer mortality in the United States. We examined the geographic differences in breast cancer incidence in Tennessee between Appalachian and non-Appalachian counties from 2005 to 2015.

**Methods:**

We used ArcGIS 10.7 geospatial analysis and logistic regression on the Tennessee Cancer Registry incidence data for adult women aged ≥ 18 years (N = 59,287) who were diagnosed with breast cancer from 2005 to 2015 to evaluate distribution patterns by Appalachian county designation. The Tennessee Cancer Registry is a population-based, central cancer registry serving the citizens of Tennessee and was established by Tennessee law to collect and monitor cancer incidence. The main outcome was breast cancer stage at diagnosis. Independent variables were age, race, marital status, type of health insurance, and county of residence.

**Results:**

Majority of the sample were White (85.5%), married (58.6%), aged ≥ 70 (31.3%) and diagnosed with an early stage breast cancer (69.6%). More than half of the women had public health insurance (54.2%), followed by private health insurance coverage (44.4%). Over half of the women resided in non-Appalachian counties, whereas 47.6% were in the Appalachian counties. We observed a significant association among breast cancer patients with respect to marital status and type of health insurance coverage (*p* =  < 0.0001). While the logistic regression did not show a significant result between county of residence and breast cancer incidence, the spatial analysis revealed geographic differences between Appalachian and non-Appalachian counties. The highest incidence rates of 997.49–1164.59/100,000 were reported in 6 Appalachian counties (Anderson, Blount, Knox, Rhea, Roane, and Van Buren) compared to 3 non-Appalachian counties (Fayette, Marshall, and Williamson).

**Conclusions:**

There is a need to expand resources in Appalachian Tennessee to enhance breast cancer screening and early detection. Using geospatial techniques can further elucidate disparities that may be overlooked in conventional linear analyses to improve women’s cancer health and associated outcomes.

## Background

**Fig. 1:**
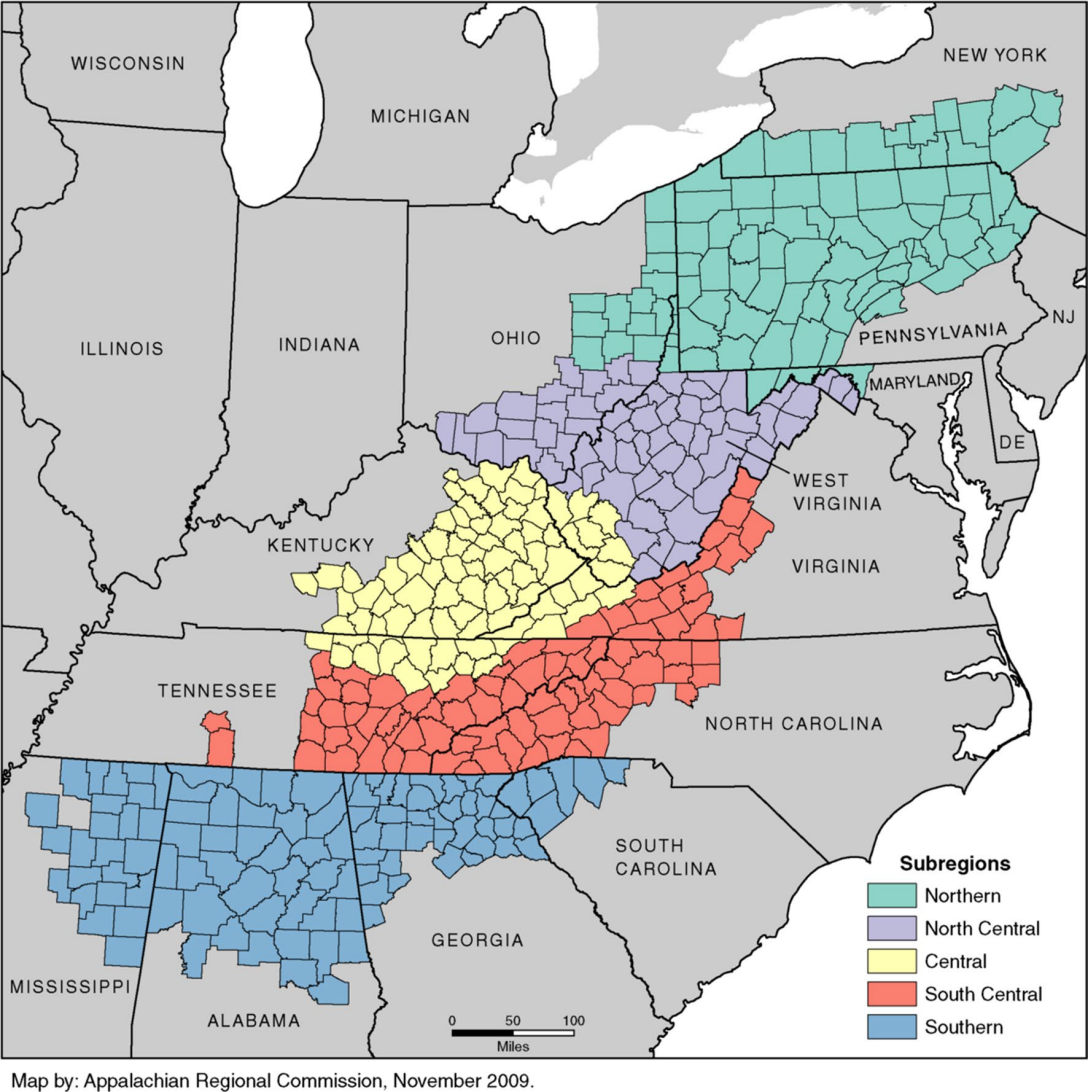
Appalachian region. Map by Appalachian Regional Commission

Breast cancer remains the most commonly diagnosed cancer, as well as the second leading cause of cancer death among women in the United States (U.S.). The burden of breast cancer, however, varies by the region (rural vs urban) where an individual lives [1, 2]. The Appalachian region is defined as the 205,000-square-mile region that stretches from southern New York to northern Mississippi, including 420 counties over 13 states [3] (see Fig. 1). One out of every three counties in the Appalachian region is considered rural. The Appalachian geography isolates many communities from cities and healthcare resources, where the residents of this region are often described as the “invisible/neglected minority” [4]. Mountain chains, valleys, and rivers often separate small communities and inhibit transportation to healthcare services [5]. Residents of the Appalachian region experience higher rates of poverty, lower levels of literacy, and poorer health outcomes compared to the non-Appalachian region [6, 7]. Although cancer mortality rates in the U.S. have continuously decreased since 1991, geographical and racial/ethnic disparities in cancer mortality and survival persist [4, 7, 8] in the Appalachian region.

Figure 1 shows the map of the Appalachian states, counties, and subregions

Central Appalachian comprises 234 counties across six states. These are Eastern Kentucky, Southwest North Carolina, Southeast Ohio, Northeast Tennessee, Southwest Virginia, and West Virginia. It is the most distressed, rural, deprived, and at-risk part of Appalachia with a disproportionately high burden of cancer [9–13] and low socioeconomic characteristics [13–15], yet, existing research rarely includes Central Appalachia [3, 16, 17]. Among many challenges contributing to this significant health disparity, evidence showed that residents of rural Appalachia are at high risk for not receiving recommended cancer screening tests, including mammograms for breast cancer [10, 18–23]. Lack of cancer screening has been attributed to lack of education, unhealthy behavioral lifestyles [7, 15, 20, 24–27], of the Appalachian culture [21, 27–32], geographical variations [10, 26, 33–35] and persistent poverty [15, 26].

Tennessee is one of the largest and most diverse states in the Appalachian region in terms of race/ethnicity, income, and location (i.e., remote, rural, small towns, rural–urban, and urban) compared to similar states that are largely rural such as Kentucky and West Virginia. Over half (52) of the counties in Tennessee are classified as Appalachia (eastern part, mainly rural & small towns) which tends to be poorer yet understudied. According to the America’s Health Rankings, Tennessee cancer death rates have increased 7% since 1990, and the state currently ranks 44th and 45th in health outcomes and cancer deaths nationally [36].

Breast cancer disparities are particularly high in Tennessee. Tennessee women experience the 12th highest breast cancer mortality‐to‐incidence ratio compared to other women in all 50 U.S. states [37]. Overall, breast cancer mortality rates are about 40% higher in Black women compared with White women [38]. Black women in Tennessee rank first in breast cancer mortality nationally [39]. Few studies have assessed cancer incidence patterns by Appalachian designation or residence in the state.

According to Penchansky et al. [40] and Gold [41], there are two dimensions of healthcare access. The first is economic access which refers to affordability and the second is geographic access which includes reasonable distance travel time to providers. Studies on geographic access also suggest that people usually seek healthcare in places that are nearer to them than those that are greater distance away [42]. Similarly, Williams et al. [43] also concluded that people may be discouraged from seeking early medical care if they are to travel distances. While the place of residence/geography alone cannot determine the risk of cancer, it can impact prevention, diagnosis, and treatment opportunities. Tennessee, in particular, has been understudied in the area of cancer disparities, notwithstanding, its unique characteristics/classification as both Appalachia and non-Appalachian state. Considering this, the goal of this analysis was to explore the geographic disparities patterns in breast cancer incidence in Tennessee by Appalachian and non-Appalachian county of residence/designation.

## Methods

### Data source and study population

The study population includes all female Tennessee residents aged ≥ 18 years diagnosed with histologically confirmed breast cancer as the primary site of diagnosis as coded by the International Classification of Diseases for Oncology, Third Edition (ICD-O-3), and reported to the Tennessee Cancer Registry (TCR) from January 1, 2005 to December 31, 2015 (*N* = 62,481). Detail information about TCR and the dataset is described at https://www.tn.gov/health/health-program-areas/tcr/tennessee-cancer-registry-data.html and in previously publication [44]. Of the total 62,481 cases, Men [n = 643 (1.3%)], other races [n = 838 (1.3%)], unknown and other missing [n = 1713 (2.7%)] information were excluded. Analysis was performed on a total of 59,278 breast cancer cases. Data used for this analysis is restricted but available by request to the Tennessee Department of Health-TCR at https://www.tn.gov/education/data/data-downloads/request-data.html. Analytical files are available by reasonable request to and approval by the Tennessee Department of Health (TDH). The Tennessee Department of Health Institutional Review Board approved the research protocol.

### Individual-level characteristics

Individual-level sociodemographic variables obtained from TCR included derived stage at diagnosis, age, race, marital status, type of health insurance, and county of residence. The main outcome stage at diagnosis was categorized as ‘early' (defined as in situ and localized stages) and 'late' (defined as regional and distant stages). Age was dichotomized as < 50; 50–59; 60–69 and ≥ 70 years. According to the U.S. Census Burau, the racial/ethnic composition of Tennessee is 73.0% White (Non-Hispanic); 16.7% Black or African American; 0.3% American Indian and Alaska Native; 1.4% Asians; 0.1% Native Hawaiian and Other Pacific Islander; some other race 2.2%; two or more races 1.7%; and 4.6% Hispanic or Latino [45]. However, due to the small number of cancer cases reported among the other racial groups (American Indian and Alaska Native; Asians; Native Hawaiian & Other Pacific Islander; & Hispanic/Latino), the TDH/TCR did not allow us to use these “other racial/ethnic” groups in the analysis to ensure privacy and confidentiality of the patients. As a result, race was limited to Black and White. Marital status was categorized as single/never married; married/common-law; divorced/separated; and widow.

Unlike other developed/industrialized countries, the U.S. does not have a universal healthcare coverage, rather the U.S. operates a hybrid system (i.e. publicly funded and private health insurance). Public health insurance is a program run by federal, state, or local governments in which people have some or all their healthcare costs paid for by the government (e.g. Medicare, Medicaid, TRICARE etc.). Medicare is a federally administered program that provides health insurance for senior citizens (age 65 +) and certain disabled people. This health insurance covers medical expenses such as doctor’s visits, hospital stays, drugs, and other treatment. Medicaid covers some low-income individuals, their families or those with disabilities who are below the national poverty line. Private insurance, the dominant form of coverage, is primarily employer-sponsored and/or paid by the individual. Private health insurance is provided by employers which requires the insured person to pay a monthly premium in addition to a co-payment when you receive healthcare. Examples include fee-for-service, Health Maintenance Organizations (HMOs) etc. Subsequently, type of health insurance coverage was categorized as public (Indian Health Service, Medicaid, Medicare, and Veterans’ Affairs including TRICARE,); private (fee for service, Health Maintenance Organization [HMO], Managed Care, and Preferred Provider Organization [PPO]); and self-pay or uninsured. Place/county of residence was categorized into two groups on whether the patient resided in an Appalachian or non-Appalachian county.

### Statistical and spatial analysis

We first obtained the distribution of continuous age variable by mean, standard deviation, and range (lower and upper age at diagnosis). Next, the characteristics of patients categorial variables were summarized using frequencies and percentages. We also assessed the association between the categorial variables using chi-square tests. Fisher’s exact tests were used to confirm chi-square tests and p-values reported (Table 1). The data was then imported into Environmental Systems Research Institute (ESRI) ArcGIS 10.7 to generate a map of all breast cancer incidence cases in Tennessee. ArcGIS is a geospatial software to view create, manage, share, and analyse spatial/geographic data. ESRI develops ArcGIS for mapping on desktop, mobile, and web. Using the 2010 Tennessee population census, age-adjusted incidence rates were calculated by county. Step by step guidance of age adjusted calculation is shown at https://seer.cancer.gov/seerstat/tutorials/aarates/step1.html. The calculated rates represent the cumulative rate for the 11-year period. To visually compare rates in non-Appalachian (n = 43) and Appalachian (n = 52) counties, a boarder was generated around the Appalachian counties. Reference points were added to visualize the five major cities in the state. Statistical analyses were conducted using SAS statistical software, version 9.4 (Cary, NC), while spatial analyses were conducted using ESRI ArcGIS 10.7.

**Table 1:** Characteristics of breast cancer patients (*N* = 59,287)

	Mean	SD	
Age at diagnosis (Range: 18, 105)	61.5	13.7	

	Number	Percent (%)	*P*-value
*Stage at diagnosis*			
Early (in situ and localized)	41,267	69.6	
Late (regional and distant)	18,011	30.4	
*Race*			0.9797
White (ref)	50,716	85.5	
Black	8571	14.5	
*Age at diagnosis*			0.9912
< 50	11,334	19.1	
50–59	14,393	24.3	
60–69	14,975	25.3	
≥ 70 (ref)	18,585	31.3	
Marital status*			** < 0.0001**
Single/never married (ref)	6859	13.0	
Married/common law	30,947	58.6	
Divorced/separated	6216	11.8	
Widowed	8814	16.6	
Type of health insurance*			** < 0.0001**
Self-pay/uninsured (ref)	690	1.35	
Public	27,630	54.2	
Private	22,648	44.4	
*County of residence*			0.8076
Non-Appalachian (ref)	31,096	52.4	
Appalachian	28,191	47.6	

Bold implies marital status and type of insurance coverage are statistically significantly associated among women diagnosed with breast cancer in Tennessee

Public Insurance (Indian Health Service, Medicaid, Medicare, and Veterans’ Affairs including TRICARE)

Private Insurance (fee for service, HMO, Managed Care, and PPO)

R, Range; SD, Standard deviation

^*^Total values not equal to full sample due to missing data

## Results

### Patients sociodemographic descriptive

Table 1 provides summary descriptive statistics on breast cancer cases diagnosed from 2005 to 2015 for all female (White and Black/African American) residents aged 18 years and older in Tennessee. Patient ages range from 18 to 105 years. The mean age at diagnosis for the whole sample was 61.5 years old with a standard deviation (SD) of 13.7. Most women in the study (85.5%; n = 50,716) were White, married (58.6%; n = 30,947), aged ≥ 70 (31.3%; 18,585), and diagnosed with an early stage of breast cancer (69.6%; n = 41,267). More than half of the women had public health insurance (54.2%; n = 27,630), followed by private health insurance coverage (44.4%; n = 22,648). Over half of the women (n = 31,096) resided in non-Appalachian, whereas 47.6% (n = 28,191) were in Appalachia. We observed a significant association among breast cancer patients with respect to marital status and type of health insurance coverage (*p* =  < 0.0001). See Table 1.

### Spatial distribution by county of residence

**Fig. 2:**
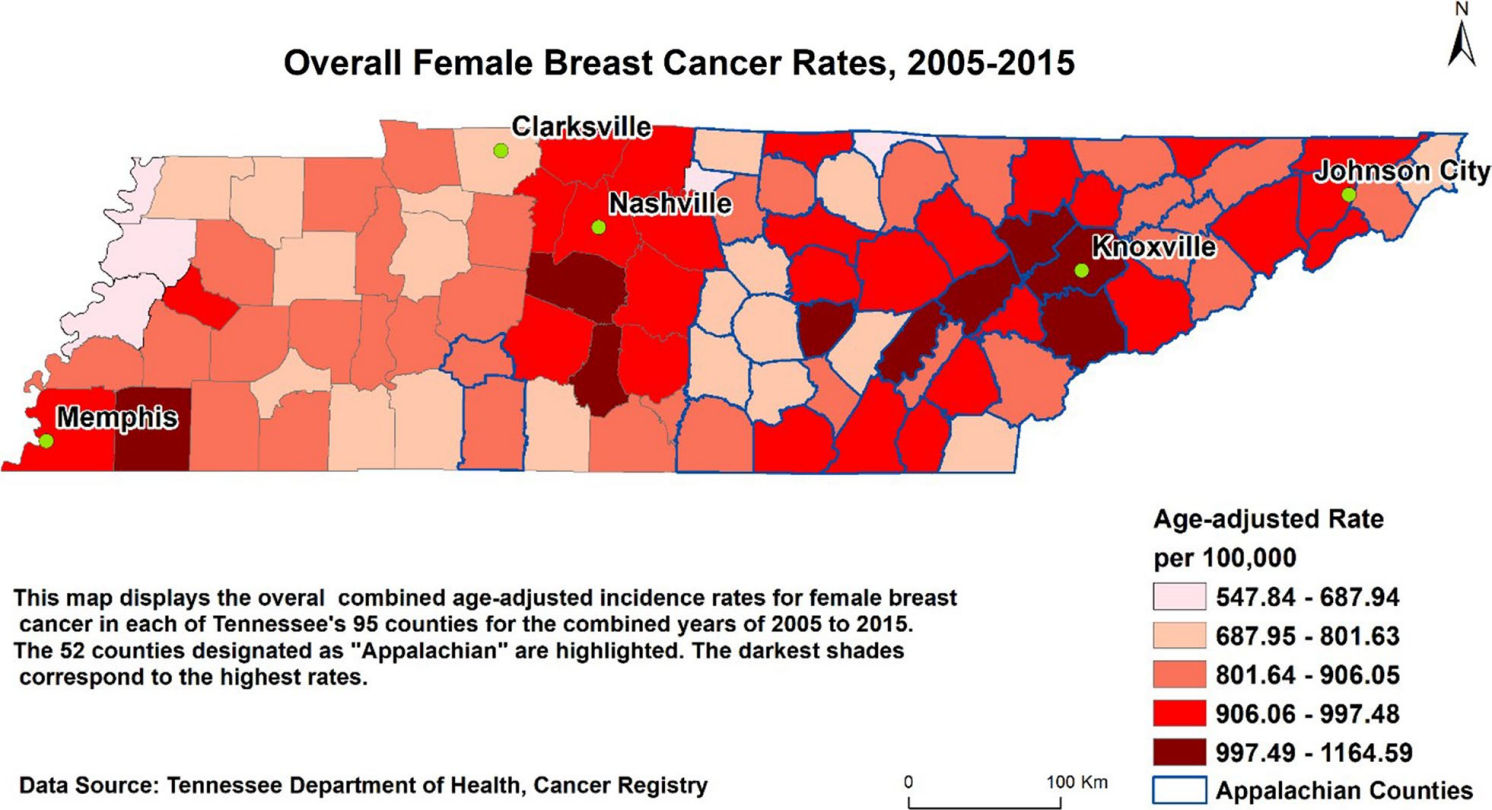
Overall female breast cancer rates, 2005–2015. Map generated by the authors

The spatial analysis map depicts the overall breast cancer incidence age-adjusted rates across the 95 counties from 2005 to 2015 (See Fig. 2). Incidence rates are displayed along a color gradient, meaning the darkest colors represent the highest rates, while the lightest the lowest. Although reported breast cancer incidences were slightly greater in non-Appalachian counties, the age-adjusted results show higher rates in the Appalachian counties compared to non-Appalachian counties. Also, we observe a clustering of mid-to-high rates around Nashville (central region), Knoxville, and Johnson City (northeast region). The highest age-adjusted rates of 997.49–1164.59/100,000 were recorded in 9 counties with 6 in Appalachia (Anderson, Blount, Knox, Rhea, Roane, and Van Buren), and 3 in non-Appalachia (Fayette, Marshall, and Williamson). To establish spatial autocorrelation, we ran a test for Moran’s index and found a correlation between the clustering of similar high and low rate counties (MI = 0.203, *p* = 0.012). Further, the map also indicated that regardless of Appalachian designation, clustering of the highest rates was reported near major cities, i.e. Johnson City and Knoxville (Appalachian), Memphis, and Nashville (non-Appalachian) (see Fig. 2).

Figure 2 shows combined age-adjusted incidence rates for female breast cancer in each of Tennessee’s 95 counties from 2005–2015

## Discussion

The Appalachian region of the U.S. represents 8% (i.e. > 25 million) of the total population [3]. The region is a National Cancer Institute-designated priority area as it represents one of the most economically disadvantaged, medically underserved regions, and experiences elevated breast cancer risk and other disproportionate cancer burdens [10, 46]. Complex and interrelated set of individual, interpersonal, community, and societal factors are known to contribute to health inequities and disparities in breast cancer incidence, diagnosis and mortality in the Appalachian region [47–49]. The goal of this snapshot was to enhance our understanding of breast cancer incidence distribution by Appalachian county of residence and determine if geographic disparities exist in Tennessee.

Age is one of the important risk factors for breast cancer, and most breast cancer cases are diagnosed in women ≥ 50 years old. However, about 11% of breast cancer cases occur in women below age < 45 in the U.S. when the disease is more aggressive leading to poorer prognosis and survival compared to older women [50]. Previously, Partridge et al. [51] examined the effect of age, and delay in breast cancer stage at diagnosis using the National Comprehensive Cancer Network (NCCN) Breast Cancer Outcomes Database Project. They found that women aged ≤ 40 had a 50% (OR 1.52; 95% CI 1.39–1.67; p < 0.0001) higher likelihood of > 60 days delay in breast cancer diagnosis than older women [51]. Another study from Sweden evaluated the relationship between age and breast cancer and found that age was positively associated with high breast cancer-specific mortality especially among women < 40 years old [52]. In the present study, between 2005 and 2015 more breast cancer cases were diagnosed in women ≥ 50 years compared to younger women under age 50. The highest number of cases was reported among women ≥ 70 years (31.3%), followed by women aged 50–59 (24.3%), and 60–69 (25.35) year, however, the association was not significant (*p* = 0.9912) among women diagnosed with breast cancer in Tennessee. There was also no significant difference between White women and Black women diagnosed with breast cancer (*p* = 0.9797) in Tennessee.

Studies have reported a lower breast cancer risk for married women than single/unmarried women [53–56]. Factors attributed to this include financial stability, engaging in physical activity, healthy behavioral lifestyles like better nutrition, family support, adherence to recommended screening and treatment [53–56]. In contrast, Barry and Breen reported that marital status and being Black both contribute to late-stage diagnosis in Atlanta, GA [57]. Specifically, in the city of Detroit, being unmarried, being Black, and residing in an area of extreme poverty were all significant [57]. Health insurance is also identified as a predictor of access and use of healthcare services [54, 58–60]. In our study, we found marital status and type of health insurance coverage played a significant role in the diagnosis of breast cancer.

Place of residence, neighborhood socioeconomic characteristics, and built environments are recognized as important contexts through which our health is shaped. Previous studies [2, 26, 61–68], reported extraordinarily high cancer rates and health disparities in Appalachia and rural America. A recent Centers for Disease Control and Prevention’s (CDC) Morbidity and Mortality Weekly Report noted that rural America experienced an increase in cancer deaths from 2006 to 2015 despite a lower incidence (2004–2013) compared to urban America [68]. Using the National Program of Cancer Registries linked with Medicare claims from Pennsylvania, Ohio and Kentucky, Anderson et al. [64], analyzed breast cancer screening, geographic deprivation, and late stage diagnosis. The authors revealed that counties with the highest economic deprivation reported the least screening, and the highest rates of late-stage diagnosis (21.05 vs 15.10 vs 17.17). Kentucky the state with the most deprived Appalachian counties, reported the strongest association compared to Pennsylvania, and Ohio counties [64]. Similarly, a systematic review that examined the relationship between place of residence and late breast cancer incidence found that almost 50% (17/36) of the eligible studies indicated breast cancer patients residing in geographically remote/rural areas had more late-stage diagnosis than urban women [2].

While our reported proportion of breast cancer incidence in the non-Appalachian counties (31,096; 52.4%) was slightly higher than Appalachian counties (28,191; 47.6%), the difference was not significant (*p* = 0.8076). On the other hand, Bennett et al. [69] analyzed the effect of urban versus rural residence on stage at diagnosis and survival among women with breast cancer in Aotearoa, New Zealand. Results showed that living in an urban or rural area did not have any statistically significant effect on breast cancer stage at diagnosis or survival [69]. However, consistent with previous findings from the U.S., and across the world [70, 71], our spatial analysis confirms geographical disparities exist between Appalachian and non-Appalachian counties in the overall age-adjusted breast cancer rates mainly around central and eastern Tennessee. Tin et al. [70] examined ethnic disparities in breast cancer survival with Māori (indigenous people) and Pacific women (immigrants or descended from immigrants from Pacific Islands women). They reported that over half of Maori and Pacific women lived in the most or predominantly deprived neighborhoods and were twice as likely to die from breast cancer when compared to non-Maori non-Pacific women [70]. Likewise, Valery et al. [71] investigated the effect of stage at diagnosis, treatment, and comorbidities on survival between non-Indigenous and Indigenous cancer patients. Results showed that compared to non-Indigenous patients, Indigenous patients in Queensland, New Zealand, were 1·5 times more likely (95% CI 1·3–1·7) to die from the disease [71].

Rural/urban disparities in breast cancer incidence/diagnosis, treatment, mortality, and survival have been reported among minority populations and other underprivileged groups around the world. Although the underlying causes for the observable breast cancer outcomes are complex and not well understood some authors have attributed it to geography, economic or political condition, limited resources and inequity in healthcare access and quality [72, 73]. For instance, many Appalachian areas are isolated from larger cities limiting ease and equitable access to required medical resources as already noted. However, Tennessee is a unique state as major metropolitan cities Johnson City, Kingsport, and Knoxville fall within the Appalachian region, providing rural/urban diversity not seen in many other Appalachia states like Kentucky and West Virginia. As a result, expanding healthcare resources in these urban Appalachian metropolitan cities may benefit the state’s Appalachian communities and enhance breast cancer screening as well as early detection, a potential factor in the increased incidence rates observed in these areas.

Our findings serve to provide important public health implications not just for the Appalachian/rural regions of the United States, but other parts of the world that share similar socioeconomic and geographic characteristics. As previously mentioned, place of residence, neighborhood socioeconomic characteristics, and built environment are major factors in shaping health. Geographic differences in breast cancer incidence found in this study can be used by local public health officials to focus prevention efforts on counties that are in need and would benefit the most from strategies aimed at preventing cancer from occurring and reduce disparities. When examining other resource-limited areas around the world, the implementation of methods used in this study can draw attention to areas of need. Anderson et al. [74] previously described the barriers to breast cancer care in limited-resource settings as being a lack or recognition of cancer as a public health priority. Additional issues such as trained healthcare personnel shortages and migration, public and healthcare provider educational deficits, and social barriers may also impede patient entry into early detection and cancer treatment programs [74]. To bridge the disparities gap, identifying the scope of the problem in underserved or limited-resource areas (i.e. the Appalachian region and similar places) is an important step in controlling and reducing barriers to breast cancer diagnosis and fatality around the world.

### Strengths and limitations

This study adds to the existing literature by using the geographic information system to explore breast cancer incidence patterns among women living in Appalachian Tennessee and non-Appalachian Tennessee counties. We found a higher overall age-adjusted incidence rate among women in Appalachian counties compared to non-Appalachian counties. This is an important finding considering the unique geographical and social characteristics of Tennessee, a state not covered in the Surveillance, Epidemiology, and End Results program (SEER) [75]. Further, as determinants of health such as environmental, socio-cultural, and physical environment differ greatly from place to place, so do people’s healthcare needs. The strength of the geospatial analysis is shown through the insignificant result of breast cancer and county of residence in t-tests. However, significantly higher cluster rates were observed among Appalachian counties using spatial analysis. We would not have been able to determine this relationship without the techniques we used. It is important to point out that rural urban differences in breast cancer incidence are not the same everywhere and may differ within countries, regions, states, or counties and should not be generalized. Therefore, the use of Geographic Information System (GIS) tools is useful to understand public health issues in any area/geography, as well as provide useful information in decision making. In particular GIS can serve as an essential tool for researchers, social scientists, health educators to understand their population. It is also a powerful tool for health planning, monitoring and the evaluation of the effectiveness of health programs aimed at reducing premature disability and death due to breast cancer, in Tennessee and in similar settings around the world.

Nonetheless, our study is not without limitation. First, we were limited by the retrospective administrative variables available to us. For instance, cancer registries do not collect socioeconomic status data (e.g., income, education, etc.), behavior/lifestyle factors (e.g. smoking, nutrition/diet, etc.), and quality of treatment received by patients. Sociodemographic variables such as age, race, insurance status, and marital status made available to use were only collected at the time of diagnosis and may not be up to date. Second, the study focused on a single state in the United States, Tennessee, which limits its generalizability to another state, geographic location, region, or country different from Tennessee. Despite, these limitations, the findings are essential since they provide a better understanding of place of residence and breast cancer disparities for Tennessean women and provide methods for establishing a baseline of geographic variation in cancer incidence.

## Conclusions

This study used Geographic Information System to investigate breast cancer incidence patterns by Appalachian county designation in Tennessee. We observed spatial disparities in the Appalachian county of residence, as well as significant relationship in marital status, type of health insurance, and breast cancer through the univariate analysis but did not show an association by age at diagnosis, race, and county of residence. As a next step, future studies will continue to utilize GIS methods to examine the relationship between the location of healthcare facilities and distance traveled (transportation) and the potential effect on cancer treatment, mortality, and quality of life. We will also link the cancer incidence-to-mortality as well as medical record data to assess survival outcomes by sociodemographic characteristics, socioeconomic status, and other lifestyle behaviors. Further, additional research should focus on other neighborhood-level factors that may impact cancer health outcomes, outside of Appalachian designation, in addition to an examination of screening trends on incidence and mortality.

## Data Availability

Data used for this analysis are restricted but available by request to the Tennessee Department of Health, Tennessee Cancer Registry (https://www.tn.gov/education/data/data-downloads/request-data.html). All analytical files are available by reasonable request and per Tennessee Department of Health approval.

## Abbreviations

CDC: Centers for Disease Control and Prevention
ESRI: Environmental Systems Research Institute
GIS: Geographic Information System
HMO: Health Maintenance Organization
ICD-O-3: International Classification of Diseases for Oncology, Third Edition
NCCN: National Comprehensive Cancer Network
PPO: Preferred Provider Organization
SD: Standard deviation
SEER: Surveillance, Epidemiology, and End Results program
TCR: Tennessee Cancer Registry
TDH: Tennessee Department of Health
VA: Veterans’ Affairs
